# Mechanisms of Brain Signaling During Sepsis

**DOI:** 10.2174/157015909790031175

**Published:** 2009-12

**Authors:** Najla Akrout, Tarek Sharshar, Djillali Annane

**Affiliations:** General Intensive Care Unit, Raymond Poincaré Hospital (AP-HP), University of Versailles SQY (UniverSud Paris), 104 boulevard Raymond Poincaré, 92380 Garches, France

**Keywords:** Sepsis, hippocampus, hypothalamus, nitric oxide, complement, prostaglandins, apoptosis.

## Abstract

Brain signaling is a crucial event for the body to mount an appropriate response to invading microorganisms. Pro-inflammatory cytokines are released from infected tissues and reach key structures in the brain *via *the circumventricular organs, areas of damaged blood brain barrier or they cross actively the blood brain barrier using specific carriers. Alternately, cytokines may activate brain endothelial cells or microglial to produce prostaglandins which then diffuse into the brain to activate neurons. Finally, cytokines may activate the autonomic nervous system at the periphery. The following crosstalk between astrocytes and microglial precedes neuronal activation particularly within the hippocampus, amygdale and hypothalamus. The resulting release of neuro-hormones in the systemic circulation allows restoration of homeostasis. It is likely that an excess in nitric oxide and complement anaphylatoxin C5a contributes to DNA damage within neurons of the hippocampus and hypothalamus and subsequent brain dysfunction.

## INTRODUCTION

Sepsis is a clinical syndrome that reflects an uncontrolled systemic inflammatory response to an infection. This syndrome usually includes a source of infection, and at least two of the four signs of a systemic inflammatory response syndrome (SIRS): fever or hypothermia, tachycardia, rapid breathing, hyperleucocytosis or leucopoenia. When the inflammatory response is sustained, patients will develop organ dysfunction, shock and may die. This disease still places a burden to the healthcare system worldwide with an annual incidence of about 200 cases per 100000 in-habitants and a mortality rate of 25%, and up to 60% when shock is present [1,52].

An altered mental status is a common feature of severe sepsis, and is termed sepsis associated encephalopathy, or sepsis associated delirium [56,58]. Indeed, the neurological manifestations may variably include delirium, agitation and coma. Sometimes brain dysfunction is more subtle, or masked by sedative drugs, and can be evidenced only using electrophysiological testing, or more cumbersome examinations for intensive care unit patients, such as magnetic resonance imaging showing structural abnormalities [58]. There is enough evidence to suggest that the more severe the encephalopathy the lower the survival chance [24,56]. 

The brain is highly protected from the rest of the body and particularly from the blood and potential circulating products. The brain plays also a major role in orchestrating host response to stress like infection. In this review we will summarize how brain is activated following infection, its subsequent normal response, and we will discuss potential mechanisms that may cause central nervous system failure and ensuing progression of inflammation to death. 

## SIGNALING PATHWAYS

The vagus nerve and circumventricular organs (CVOs) are the two main pathways to signal the brain during inflammation and sepsis whereas peripheral cortisol exerts a negative feedback. 

The vagus nerve is a sensor of visceral inflammation through its axonal cytokine receptors. It is comprised of non-myelinated fibers with free nerve endings. These terminal parts of the vagus nerve are sensitive to mechanical states of the viscera and local changes in the chemical environment. Afferent vagal fibers terminate in the nucleus tractus solitarius which controls the baroreflex and is connected to other autonomic structures and to paraventricular nuclei of the hypothalamus. 

The circumventricular organs are discrete central nervous system sites that lack a blood brain barrier. Some of them are located in the vicinity of neuroendocrine structures (e.g., the neurohypophysis, vascular organ of the lamina terminalis, subfornical organs, pineal gland, and subcommissural organ), others are close to brainstem autonomic centers (i.e., area postrema). The circumventricular organs express components of innate and adaptative immune systems, such as toll-like receptors, CD14, and cytokine receptors including receptors to interleukin (IL) 1β, IL 6, and tumor necrosis factor (TNF) a αpro-inflammatory cytokines that induce fever, activate the neuroendocrine and autonomic centers) [15]. 

## KEY BRAIN SYSTEMS INVOLVED IN THE RESPONSE TO SEVERE INFECTIONS

**Fig. (1):**
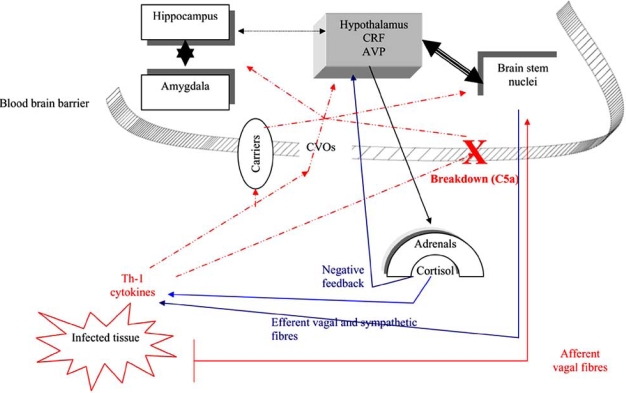
Key structures involved in brain signalling during severe infections. Invading microorganisms result in release of pro-inflammatory cytokines that enter the brain through CVOs lacking a blood brain barrier, through areas where the blood brain barrier is breakdown, or *via* active transport across the blood brain barrier. In addition, afferent vagal fibres are activated *via* cytokines receptors at the level of peripheral tissue. Red colour indicates activating signals to the brain. Blue colour indicates negative controls. BBB CVOs = circumventricular organs. CRF= corticotrophin releasing factor – AVP = vasopressin.

Three main systems are tightly interconnected to orchestrate homeostasis in stressful conditions such as sepsis, trauma or surgery. These structures include: the limbic system (mainly the hippocampus and amygdala), the hypothalamic-pituitary axis and the Locus Coeruleus / noradrenergic system (Fig. **1**).

The limbic system is comprised of four main structures, the amygdala, the hippocampus, the limbic cortex and the septal area. The amygdala is connected to the hippocampus, septal nuclei, and prefrontal areas and to the medial dorsal nucleus of the thalamus. It plays a major role for the identification of danger and self preservation. Stimulation of the amygdala results in anxiety and fear, with alertness and a stage of being ready to fly or fight. Inhibition of amygdala neurons renders the body indifferent to danger. The hippocampus is mainly responsible for long term memory; it allows the comparison of present stress with similar past experiences, enabling the choice of an appropriate response to the stress for surviving. In animals, hippocampus lesions may result in thymic atrophy and decreased lymphocytes counts suggesting immune-suppression [10]. Activation of the hippocampus suppresses corticotropin releasing hormone (CRH) synthesis [61]. Because no direct anatomical substrate between hippocampus/cortex and hypothalamic parvocellular neurons have yet been successfully identified, it is likely that cortico-hippocampal influences on hypothalamic hypophysiotropic neurons are indirectly achieved *via* subcortical relay neurons. The hypothalamus is organized into three regions including the lateral, medial and periventricular hypothalamus, each having distinct morphological and functional features. The paraventricular nuclei are localized in the periventricular hypothalamus. It is organized into three cellular divisions: a medial group that produces CRH and releases it into the hypophysial portal system; an intermediate group that secretes vasopressin in association with the supra optic nuclei to be stored in the posterior pituitary gland; a lateral group that produces CRH and innervates noradrenergic neurons in the brain stem. Hypothalamic derived peptides that stimulate the pituitary gland include CRH, LH-releasing hormone, FSH-releasing factor, GH-releasing factor, prolactin stimulating factor and thyrotropin –releasing hormone. Other peptides are inhibiting factors like GH-inhibiting hormone (somatostatin) and PRL-inhibiting hormone. Vasopressin, natriuretic peptides, and catecholamines also influence the pituitary function by direct action on the gland. The effect of CRH on ACTH release by the pituitary is permissive and vasopressin acts in synergy with CRH. There are tight interconnections between projections of CRH –synthesizing neurons from the parvocellular nuclei to the brain stem and reciprocally noradrenergic projections originating from the Locus Coeruleus, located in the rostral pons and controlling arousal and cardiovascular autonomic centers, and ending with synapse on cholinergic inter-neurons in the parvocellular nuclei. Thereby, noradrenaline, CRH and vasopressin can stimulate each other. Through collateral fibers, ultra short negative feedback loops allow permanent adaptation of the synergy between the two systems [12]. Finally, CRH, vasopressin and noradrenaline are on the stimulatory control of the serotoninergic, cholinergic and histaminergic systems and are inhibited by the gamma amino butyric acid, benzodiazepine and opioids systems. The limbic, hypothalamic, and noradrenergic systems fine-tune each other to maintain homeostasis in presence of a stressor.

## CYTOKINES TRAFFICKING TO THE BRAIN

Invading microorganisms are recognized by immune cells through a number of molecular pattern recognition molecules. The subsequent activation of immune cells results in the release of pro-inflammatory cytokines within the focus of infection and in the arterial circulation [1]. It is now recognized that increase in cytokine concentrations, e.g. IL-1, in the periphery increases the turnover of noradrenaline in the hypothalamus [16,17] and increases peripheral plasma [4] and brain [16] noradrenaline metabolism and extracellular levels. Similarly, intracerebroventricular and peripheral injection of interferon (IFN)-α or IL-1β produces a sustained increase of the sympathetic activity of the splenic nerve and increases the turnover of NE in the spleen [30]. Brain expression of pro-inflammatory cytokines has been shown in both animal [64] and human [55] sepsis, particularly in the hippocampus, hypothalamic and autonomic nuclei. Yet, the size of the various cytokines is sufficient to prevent their entry into the brain by passive diffusion [11]. 

Three main mechanisms for cytokine entry into the brain have been described. Firstly, the inflammatory mediators may enter passively into the brain *via* areas lacking a blood brain barrier (BBB), mainly the CVOs. Then, they progress in deeper brain regions, mainly hypothalamic nuclei, the hippocampus, the amygdala and autonomic nuclei in the brain stem. Secondly, cytokines may actively cross the BBB *via* specific carriers, particularly in vessels neighboring hypothalamic nuclei and the Locus Coeruleus. Alternately, the neurons from the parvocellular and arcuate nuclei may have projections to the median eminence and may express LPS receptors on their surface [41]. LPS can also be carried by the cerebrospinal fluid to the third ventricle where it crosses the ependyma or acts on projections from parvocellular neurons. Active transport into the brain has been demonstrated for IL-1 [2], TNF [26] or IL-6 [51]. These cytokines cross the BBB *via* receptor mediated endocytosis, a transport that is likely regulated by both tissue and soluble factors. The transport of cytokines across the BBB is upregulated during inflammation, albeit only about 1% of circulating cytokines is effectively concerned. Studies using knocked out mice demonstrated that TNF transport across the BBB is almost entirely dependent on its receptors TNFR1 and TNFR2 [46]. While TNFR2 has a greater cell surface binding than TNFR1, the latter induced faster endocytosis. In addition, TNF associated with TNFR1 showed less degradation than with TNFR2, and may persist intact in cell’s cytosol for at least 1 hour. This cytokine is also transported by specific carriers. However, only 16% and 50% of the intact cytokine are delivered to the cerebrospinal fluid and brain parenchyma, respectively [3]. Yet, this relatively small amount of IL-6 is biologically active and contributes to brain ionic homeostasis [60]. IL-6 secreted by astroglial cells regulates Na-K-Cl cotransport in brain microvessel endothelial cells. While the BBB lacks permeation of iv TGF-β_1_, TGF-β_2_ can cross the BBB after iv administration, *via* a saturable blood-to-brain transport system [42]. After transcytosis, it is distributed throughout the brain, with the highest concentrations in the hypothalamus. Altogether, these findings suggest a major role of the BBB in regulating brain inflammation. There is a body of evidence that sepsis is associated with a breakdown of the BBB [47], which will ease the entry into the brain of pro-inflammatory mediators and other neurotoxic molecules (e.g. urea). The breakdown of the BBB is likely mediated by IL-1, TNF and NO [39].

Cytokines such as IL-1 can signal the central nervous system through stimulation of the vagus nerve and activation of brainstem regions such as the nucleus of the tractus solitarius [6,63]. Intraperitoneal injecton of bacterial products such as endotoxin elicited a brain response *via* the subdiaphragmatic vagus. Indeed, increased in cFos within sensory vagal neurons followed the endotoxin challenge, and vagotomy prevented fever and hyperalgesia induced by endotoxin, TNF or IL-1. This efferent activity in the vagus nerve, termed ‘cholinergic anti-inflammatory pathway’, releases acetylcholine in the vicinity of macrophages within the reticulo-endothelial system and leads to cellular deactivation and inhibition of cytokine release [8, 62]. The sympathetic and parasympathetic systems are thought to work hand-in-hand to modulate inflammatory responses.

Finally, cytokines like IL-1, IL-6 and TNF may activate cerebral endothelial cells or the microglial (*via* a cycloxygenase II dependent mechanism) to produce prostaglandin E2 which can enter into the brain [35]. In particular, prostaglandin E2 may activate preoptic nuclei and induce fever [20]. 

## CELLS ACTIVATION

Microglial cells activation is the hallmark of brain inflammation [7, 59], and has been documented in sepsis [9]. Circulating pro-inflammatory cytokines are major activators of microglial cells and astrocytes [22,48]. Cytokines like IL-1 or TNF injured neurons with subsequent microglial activation, or may also directly activate these cells, which in turn produce pro-inflammatory cytokines, causing a vicious circle [57]. Typically, microglial cells activation follows a temporal pattern, expressing different markers allowing them to first proliferate, then migrate to injured neurons, the so-called synapses stripping, and the nearby parenchyma, where they may act as phagocytes, and then they downregulate themselves [29]. Astrocytes are also activated by the pro-inflammatory cytokines, *via* calcium dependent mechanisms [5] and express a number of factors such as signaling receptors, growth factors and extracellular matrice proteins that are important in tissue repair [19]. TGFβ1 is constitutively expressed in normal adult brain [37]. This cytokine is expressed by parenchymal microglial cells and exerts a trophic and anti-inflammatory effect in the adult CNS [38]. TGFβ1 interferes with the early phase of microglial activation, decreasing cells proliferation and migration, without affecting their phagocytic capacity [38]. The crosstalk between microglial cells and astrocytes is paramount in regulating brain inflammation and communication with neurons [32]. Indeed, exposure of cultured astrocytes to LPS or to TNF and IL-1 activated microglial and caused a dual modulation of connexin43 channels properties. Subsequently, astrocytes gap junctional communication released active molecules that can communicate with neurons [49]. 

The cytokines induced interaction between microglial cells and astrocytes, activated  hypothalamic neurons to produce and release cortisol, vasopressin and catecholamines into the circulation, the main stress hormones to restore homeostasis during infection.

## ROLE OF NITRIC OXIDE

The synthesis of NO *via* the inducible isoform of the NO synthase (iNOS) seems to be preceded by IL-1 expression in the brain. Interestingly, i.p. injection of LPS induces IL-1β followed by iNOS mRNA within two hours, peaking in four to six hours and then returning to basal values by 24 hours [41]. The induction of IL-1β  and iNOS occurred in this study in the meninges, areas lacking a blood-brain barrier, and also in the parvocellular nuclei and the arcuate nucleus which contain the hypothalamic-releasing and –inhibiting hormone producing neurons. A temporal and spatial expression of IL-1 and iNOS in the CVOs and adjacent structures was also demonstrated [31]). Thus, it is likely that delayed synthesis of NO through iNOS activation prolongs the synthesis of hypothalamic hormones induced by LPS [41]. Similar over-expression of iNOS has been evidenced in walls of vessels neighboring the parvocellular nuclei in patients who died from septic shock [54]. Nitric Oxide behaves as a neurotoxic effector [18], and was associated with microglial cells and neuronal apoptosis in the hypothalamic and autonomic nuclei in both animals [43]) and patients who died from septic shock [54]. Subsequently, over-expression of iNOS may result in decreased CRF and ACTH [36], and vasopressin synthesis [25]. Similarly, excess in NO *via* iNOS induced apoptosis in autonomic brain nuclei may be responsible for the loss in cardiovascular variability in septic shock [54]. Finally, cytokines *via* activation of GABAergic neurons block NO induced LHRH but not FSH release, inhibit GHRH release, and stimulate somatostatin and prolactin release [40]. The regulatory action of NO is mediated by the combined activation of guanylate cyclase, cyclooxygenase and lipoxygenase [50].

Nitric oxide also blocks the mitochondrial respiratory chain (complex I and IV), inducing oxidative stress with the formation of peroxinitrite that can trigger premature neuronal death [13]. This mechanism of cell death is exacerbated by hypoxia and hyperglycemia and may be prevented by the 70-kDa heat shock proteins [34]. The DNA damage following systemic LPS challenge are particularly important within the CAP and inner blade region of the hippocampus and the hypothalamus [27]. However, NO might not be able to diffuse into brain parenchyma far from vessels and thus its role in brain dysfunction following LPS or sepsis might be limited.

## ROLE OF COMPLEMENT

The complement anaphylatoxin C5a is constitutively expressed in neuronal and non neuronal brain cells [45]. Following endotoxin administration, the C5a receptor became up-regulated in a time dependent manner within the cerebral endothelium, then microglial cells neighbouring the endothelium and finally in deeper brain parenchyma. The complement activation has been demonstrated in numerous inflammatory and degenerative, acute and chronic, diseases of the brain [44]. Following C5a up-regulation, microglial cells are recruited and activated to release pro-inflammatory cytokines, and their phagocytosis capacity is enhanced, and astrocytes are activated as well [23]. Subsequently, C5a contributes to the activation of the stress system. Indeed, systemic blockade of C5a reduced LPS-induced neuronal activation in the paraventricular nuclei and amygdala [14], and almost fully blunted the pituitary response to peritoneal sepsis [21]. Up regulation of C3 induced a breakdown in the BBB and increased gliosis, water content, up regulated TLR-4 with subsequent alterations in TNF, iNOS and aquaporin 4 [28]. Extending these findings, systemic administration of an anti-C5a neutralising antibody prevented the damage to the BBB [21]. Both blocking C5a or its receptor [53], and inhibiting the alternative complement pathway, attenuates neuronal death in experimental traumatic brain injury [33].

## CONCLUSION

The mechanism of brain signaling during sepsis is complex and still poorly understood. Following an infection, homeostasis depends on a tight interaction between the limbic system which contributes to the recognition of the danger, and the hypothalamic pituitary axis and the noradrenergic system which directly modulate metabolic, immune and hemodynamic responses. The exact mechanisms allowing these systems to recognize a peripheral inflammation are not fully known and involve the pro-inflammatory cytokines IL-1 and TNF trafficking across the blood brain barrier, *via* vagal route or *via* the circumventricular organs. While these cytokines are crucial in eliciting an appropriate stress response, they trigger the synthesis of the inducible nitric oxide synthase, which may act as a neurotoxic effector impairing the neuroendocrine response. These processes act *via* autonomic, neurohormonal and humoral structures and pathways, highly interconnected to develop an adapted response to a septic state, resulting in a balance between pro and anti inflammatory response. When this balance is broken with a dominating inflammatory response, these processes affect endothelial cells, microglial cells and neurons, inducing blood-brain barrier breakdown, dysfunction of intracellular metabolism (mitochondrial dysfunction, and oxidative stress) and cell death.
